# Nanotechnology in Cosmetics and Cosmeceuticals—A Review of Latest Advancements

**DOI:** 10.3390/gels8030173

**Published:** 2022-03-10

**Authors:** Vaibhav Gupta, Sradhanjali Mohapatra, Harshita Mishra, Uzma Farooq, Keshav Kumar, Mohammad Javed Ansari, Mohammed F. Aldawsari, Ahmed S. Alalaiwe, Mohd Aamir Mirza, Zeenat Iqbal

**Affiliations:** 1 Nanotechnology Lab, School of Pharmaceutics Education and Research (SPER), Jamia Hamdard University, New Delhi 110062, Delhi, India; vaibhavggn@yahoo.com (V.G.); sibanee@gmail.com (S.M.); uzma411@gmail.com (U.F.); keshav9225@gmail.com (K.K.); 2 Smart Society Research Team, Faculty of Business and Economics, Mendel University, 61300 Brno, Czech Republic; harshitamishra1088@gmail.com; 3 Department of Pharmaceutics, College of Pharmacy, Prince Sattam Bin Abdulaziz University, Alkharj 16278, Saudi Arabia or mj.ansari@psau.edu.sa (M.J.A.); moh.aldawsari@psau.edu.sa (M.F.A.); a.alalaiwe@psau.edu.sa (A.S.A.)

**Keywords:** nanotechnology, nanomaterial, cosmetics, cosmeceuticals, nanocosmetics, nanocosmeceuticals, patent, regulation, health hazards, toxicity

## Abstract

Nanotechnology has the potential to generate advancements and innovations in formulations and delivery systems. This fast-developing technology has been widely exploited for diagnostic and therapeutic purposes. Today, cosmetic formulations incorporating nanotechnology are a relatively new yet very promising and highly researched area. The application of nanotechnology in cosmetics has been shown to overcome the drawbacks associated with traditional cosmetics and also to add more useful features to a formulation. Nanocosmetics and nanocosmeceuticals have been extensively explored for skin, hair, nails, lips, and teeth, and the inclusion of nanomaterials has been found to improve product efficacy and consumer satisfaction. This is leading to the replacement of many traditional cosmeceuticals with nanocosmeceuticals. However, nanotoxicological studies on nanocosmeceuticals have raised concerns in terms of health hazards due to their potential skin penetration, resulting in toxic effects. This review summarizes various nanotechnology-based approaches being utilized in the delivery of cosmetics as well as cosmeceutical products, along with relevant patents. It outlines their benefits, as well as potential health and environmental risks. Further, it highlights the regulatory status of cosmeceuticals and analyzes the different regulatory guidelines in India, Europe, and the USA and discusses the different guidelines and recommendations issued by various regulatory authorities. Finally, this article seeks to provide an overview of nanocosmetics and nanocosmeceuticals and their applications in cosmetic industries, which may help consumers and regulators to gain awareness about the benefits as well as the toxicity related to the continuous and long-term uses of these products, thus encouraging their judicious use.

## 1. Introduction

Nanotechnology and nanodelivery systems are innovative areas of science that comprise the design, characterization, manufacturing, and application of materials, devices, and systems at the nanoscale level (1–100 nm). Nanotechnology, being recognized as one of the revolutionizing technologies, is extensively studied in the area of cosmetics and cosmeceuticals [1,2]. The incorporation of nanotechnology has led to advancements in cosmetic science, resulting in increased consumer demand throughout the world [3]. Presently, nanomaterials are attracting attention in this area, as they offer greater advantages over traditionally used cosmetic products. Further, the amalgamation of nanomaterials has greatly contributed to the global increase in the market share of pharmaceuticals and cosmetics. In the year 2019, the international market size of nanomaterials was estimated to be USD 8.5 billion and is expected to increase with up to a 13.1% compound annual growth rate from the years 2020 to 2027 [4]. Although the concept of nanomaterials (gold and silver nanoparticles) has been used in cosmetics for several years, the extensivity of applications has intensified in recent years.

Cosmetics are preparations that have been used by humans for a long time, primarily for regenerative purposes, and are appreciated by both genders. They can be defined as preparations that are typically used externally and can be formulated from a single or combination of substances obtained from either natural or artificial sources [5]. The US Food and Drug Administration (USFDA) defines cosmetics as a formulation “intended to be applied to the human body for cleansing, beautifying, promoting attractiveness, or altering the appearance without affecting the body’s structure or functions”. This expansive definition encompasses any material proposed for use as a component of a cosmetic item, although soap is explicitly excluded from this class [6]. However, under this act, the word “cosmeceutical” has no definition. As per the Federal Food Drug and Cosmetic Act (FD&C Act), there is no such word as “cosmeceutical”. This word is only used for industrial purposes to refer to cosmetic products with therapeutic actions. The European Union Cosmetics Directive (EUCD) defines cosmetics as “any substance or preparation intended to be placed in contact with the various external parts of the human body (epidermis, hair system, nails, lips and external genital organs) or with the teeth and the mucous membranes of the oral cavity with a view exclusively or mainly of cleaning them, perfuming them, changing their appearance and/or correcting body odours and/or protecting them or keeping them in good condition” [7]. The Drugs and Cosmetics Act 1940 and Rules 1945 defines a cosmetic as “any article intended to be rubbed, poured, sprinkled or sprayed on, or introduced into, or otherwise applied to the human body or any part thereof for cleansing, beautifying, promoting attractiveness, or altering the appearance, and includes any article intended for use as a component of cosmetic” [8]. Despite these definitions, the legal meaning of cosmetics in many nations is more extensive. In some Western nations, cosmetics are normally interpreted as just beautifying products, such as lipstick, mascara, eyeliners, highlighter, and a few other items of this kind [9].

Cosmeceuticals can be described as preparations that contain therapeutically active ingredients that specifically possess remedial effects upon surface application with traditionally used cosmetics. These products have quantifiable restorative effects on the skin and hair and are utilized for the treatment of different conditions, such as damaged hair, wrinkles, photoaging, skin dryness, light spots, hyperpigmentation, etc. Acting as a bridge between drugs and beauty care products, they promise an improvement in appearance [10,11]. Presently, cosmeceuticals are considered one of the fastest-growing segments of the personal care industry, and the market for individual consideration is massively expanding [2]. It is one of the most rapidly developing ventures, demanding an expansion in nanocosmeceuticals research, exploration, and applications.

**Figure 1:**
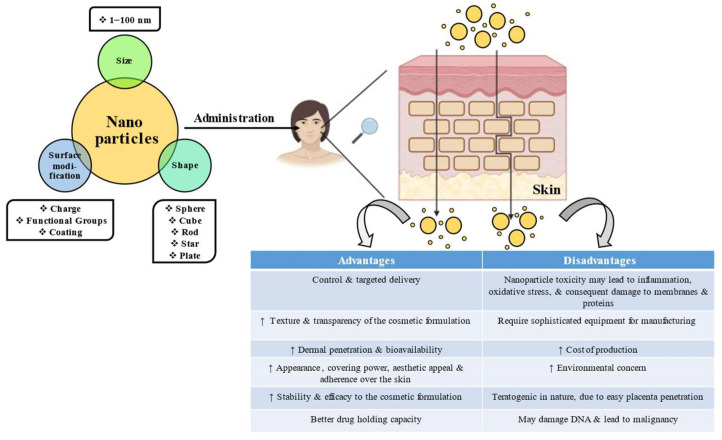
Advantages of nanocosmeceuticals.

The manipulation of materials at the atomic level by utilizing nanotechnology has great potential in the area of cosmeceuticals, opening up new avenues for the cosmetics industry. The incorporation of various nanomaterials during the development of cosmetic/cosmeceutical products results in nanocosmetics/nanocosmeceuticals, respectively. Prolongation of action, augmented bioavailability, and improved aesthetic appeal of products are a few of the advantages associated with nanotechnology-based cosmeceuticals. These products offer several other benefits over traditionally used cosmeceuticals, such as small size and huge surface-to-volume ratio, which makes them effective adjuvants in cosmeceuticals. Further, the inclusion of nanoparticles in cosmetic formulations does not change the properties of cosmeceuticals but improves their appearance, coverage, and adherence to the skin. Cosmetic manufacturers employ nanosized ingredients to improve UV protection, skin penetration, color, the release of fragrance, finish quality, anti-aging effect, and a variety of other properties. They prolong the duration of action by either controlling the delivery of active ingredients, causing site-specificity, improving biocompatibility, or enhancing the drug-loading capacity. All of these factors make them more popular among consumers, necessitating clinical trials in this area to address their safety concerns. Nanocosmeceuticals have also been highly exploited for formulating various anti-aging formulations. They are successfully marketed as skincare, hair care, and nail care products, among others, claiming to stimulate their growth, protect their structure, and increase hydration power, thus improving their effectiveness as cosmetic products [12,13]. Although they have several benefits, at the same time, they possess limitations related to stability, scalability, toxicity, cost, etc. Moreover, the safety and toxicity profiles of nanomaterials are still debatable. The small size, increased surface area, and positive surface charge of nanoparticles improve their ability to interact with the microenvironment biologically. On the other hand, they have dose-dependent toxicity through different routes of administration. It is well known that the bioavailability of an active ingredient is better influenced by the dosage rather than the physicochemical properties of the active moiety [14]. Hence, in the case of cosmetic products, a major concern in the advancement of nanoformulations is that they may enhance the concentration of active ingredients reaching the blood and impact the toxicity [15]. Figure 1 depicts the overall action of nanoparticles in cosmetics and cosmeceuticals.

Based on these facts, a narrative review of all of the relevant articles and reports was conducted by searching related keywords across different sources. Google Patents was used to collect data regarding related patents. Selected studies were compared and condensed to obtain a qualitative output based on existing theories and principles. The present review outlines various nanoparticles and nanodelivery systems used for cosmetic and cosmeceutical products, highlighting their positive and negative characteristics along with related patents. It also discusses the health and environmental risks linked with nanocosmeceuticals with suggested solutions. Further, the present review highlights the regulatory scenarios and compares the various regulations related to cosmetics. Additionally, it is intended to assist the industry and other stakeholders in identifying potential safety issues associated with nanomaterials in cosmetics. It also discusses various guidelines and recommendations prescribed by different regulatory agencies. Finally, this article aims to provide an overview of nanocosmetics and nanocosmeceuticals and their applications in cosmetic industries and suggest future directions, which may help consumers and regulators to gain awareness about the benefits as well as the toxicity related to the continuous and long-term uses of these products, thus encouraging their judicious use.

## 2. Nanomaterials Used in Cosmetic Products

Nanomaterials are materials having at least one dimension in the nano range and significantly distinct physicochemical properties. These materials have been commonly used in the cosmetic industry for many years. Cosmetics incorporating nanomaterials show more advantages as compared to microscale cosmetics. The large surface area of these particles is responsible for their efficient transportation, absorption, bioavailability, and transparency and the sustained effect of the product. However, consideration should be given to the concentration to circumvent the associated toxicity. The following Table 1 describes different nanomaterials used in the cosmetic industry.

### 2.1. Inorganic Particles

**Figure 2:**
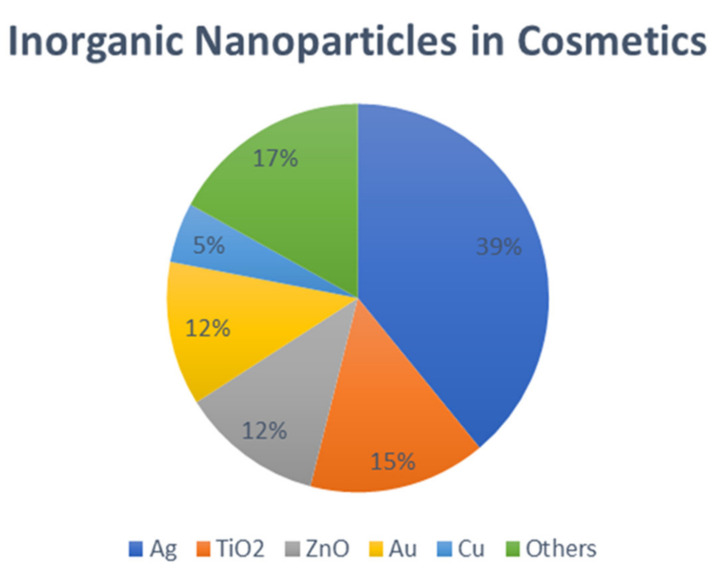
Proportions of different inorganic nanoparticles in cosmetics formulation.

These are more hydrophilic, more biocompatible, safer, and exceptionally more stable particles as compared to natural nanoparticles. They can be significantly distinct, as these nanoparticles are derived from inorganic components (Ag, Au, Ti, etc.), while the natural ones are manufactured from polymers. Figure 2 shows the percentages of different inorganic nanoparticles in cosmetic and cosmeceutical formulations.

There are many inorganic nanoparticles used in cosmeceuticals. A few important particles are described below.

#### 2.1.1. Titanium Dioxide and Zinc Oxide

Sunscreens are useful for shielding the skin from the hazardous impacts of solar radiation, including UVB, UVA-2, and UVA-1 [27,28]. They usually consist of zinc oxide (ZnO) and titanium dioxide (TiO_2_) as inorganic UV radiation filters, which prevent the harmful radiation of sunlight from reaching the skin. It has been established that ZnO is more effective for obstructing UVA, and TiO_2_ is better for the UVB range. Hence, the appropriate proportion of the mixture of these particles guarantees wide-range UV protection [29]. TiO_2_ is possibly the most broadly utilized and efficient inorganic nanoparticle for sunscreens and has a higher sun protection factor (SPF) at the nanoscale, which makes it more effective and results in a superior restorative effect due to its transparency, in contrast with its original color. These properties of TiO_2_ are attributable to its large surface-area-to-volume ratio in the nano range [30], as it makes it be highly capable of carrying molecules when their sizes are reduced to 10–20 nm. Further, it has been reported that nanoscale TiO_2_ and ZnO show incredible benefits over numerous materials that are larger than the nano range [31]. TiO_2_ and ZnO nanoparticles used as UV filters in sunscreens [32] start at a size of 20 nm. They show better scattering and produce a superior restorative or protective effect. On the other hand, inhalation of a large amount of these nanoparticles has been shown to be harmful [33]. Thus, an alternate route of administration (i.e., dermal application) focuses on normal sunscreen ingredients, as these are safer, and there is no evidence of their infiltration into the epidermis or significant toxicity issues [16,34]. The International Agency for Research on Cancer (IARC) classifies TiO_2_ as an IARC Group 2B carcinogen [32]. An investigation was carried out on rodents exposed to large quantities of TiO_2_ nanoparticles and pigments, which resulted in cellular breakdown in the lungs of the rodents; this situation is comparable to working in dusty environments, causing serious harmful effects in individuals exposed to them. However, ZnO is considered a safe entity by the USFDA for use as a UV filter in cosmetics or cosmeceuticals. As an alternative, naturally occurring nanoparticles, such as ivy nanoparticles, which are secreted from the roots of English ivy (Hedera helix), are generally safer and employed for their UV-protective effects [35]. The enhanced visual transparency and safety of Ivy nanoparticles make them an attractive alternative to replace other toxic nanoparticles, reducing the impact on the health and environment.

#### 2.1.2. Gold and Silver Nanoparticles

Gold and silver nanoparticles display antibacterial as well as antifungal properties [36] and are widely utilized in cosmetic formulations such as antiperspirants, anti-aging creams, and face masks. Gold has a long history of usage for skin health management and beauty care products in Egypt, where gold was used to maintain skin complexion. Egyptians believed that gold improved their skin composition and flexibility. Currently, gold is incorporated into different skincare items, such as salves, creams, and skincare treatments. Generally, gold in skincare products is called colloidal gold or, more precisely, nanogold if it is in the size range of 5 nm to 400 nm. Its color ranges from red to purple, depending upon the size and total surface area [2,37]. Gold nanoparticles have diverse shapes, such as nanospheres, nanorods, nanoclusters, nanostars, nanoshells, nanocubes, and nanotriangles, and the state of these particles determines their cell uptake and optical behavior. Properties such as stability and biocompatibility make them more appropriate for skincare and cosmetics [2]. Furthermore, their antifungal, antibacterial, and anti-aging benefits are well established, which are highly significant in cosmeceutical industries and in wound healing applications [38]. Gold nanoparticles play a substantial role in fixing skin damage and improving skin surface, grace, and flexibility. The soothing properties of gold make it an exceptional agent for treating skin inflammation, sunburn, and hypersensitivity. Hence, it can be successfully used in face masks and other cosmetics.

Silver nanoparticles can be utilized as successful inhibitors of various microorganisms. Silver and silver-based mixtures can be utilized to control bacterial development in different formulations [39]. The utilization of silver in cosmetics can be problematic, as silver readily precipitates in silver-based mixtures, which can be overcome by the utilization of silver nanoparticles. In Europe, the safety of colloidal silver in nanostructures concerning its use in oral and dermal cosmetic items is ambiguous [40]. In the USA, due to the lack of FDA regulations, cosmetic items are thought to lack promising antibacterial properties [41]. According to research, the use of silver nanoparticles as an additive in cosmetics makes the formulation stable, without showing sedimentation, for more than 1 year. Furthermore, silver nanoparticles showed adequate protection against microbes and their growth and did not enter human skin [42].

#### 2.1.3. Silica (SiO_2_)

Because silica nanoparticles have hydrophilic surfaces favoring extended distribution and low manufacturing costs, interest towards these materials has increased, particularly in the cosmetic sector. Nanosilica is utilized to improve the adequacy, surface, and period of actual usability of cosmetic items [43]. It has been shown that silica nanoparticles may help to improve the appearance and appropriation of shades in lipsticks and keep colors in place [44]. Silica nanoparticles are present as nanodispersions with a size range of 5 to 100 nm and can deliver both hydrophilic and lipophilic entities to their respective targets by encapsulation [12]. These nanoparticles are generally found in leave-on and wash-off cosmetic items for hair, skin, lips, face, and nails, and the further expansion of silica nanoparticles in cosmetic items is expected [45]. However, the practical uses of silica-based nanoparticles are questionable and raise concerns about their safety, but factors such as size and surface changes ought to be considered while surveying its toxicity [45,46]. However, the commercial use of silica nanoparticles in beautifying agents is still ambiguous, requiring long-term trials [16].

#### 2.1.4. Carbon Black

Carbon black, CI 77266, is known to be a significant ingredient in cosmetic formulations and is frequently utilized as a colorant in eye and skin cosmetic products. The EU has approved it for use in its nanostructure form and as a colorant at a maximum percentage of 10%. An evaluation of carbon black nanoparticles showed that they displayed a higher propensity for causing cytotoxicity, aggravation, and changes in phagocytosis in human monocytes as compared to micron-sized nanoparticles [47]. As per the EU, it can be utilized in cosmetic items when there is no danger of being breathed in [16].

#### 2.1.5. Nano-Hydroxyapatite

Nano-hydroxyapatite is utilized in cosmetic items specifically meant for oral preparations that are used for treating extreme dental sensitivity and polish remineralization of the teeth [48]. It is regarded as a promising and safe option for these purposes by the US Food and Drug Administration (USFDA) [49]. These particles have been incorporated into oral formulations, such as dentifrices and mouthwashes, owing to their remineralization and desensitization properties. Such preparations could provide an alternative to fluoride toothpaste [16].

### 2.2. Nano-Organic (Tris-Biphenyl Triazine)

Tris-biphenyl triazine is a novel, powerful, and photostable filter specifically used in sunscreen formulations [50]. In its nano form, it functions as a broad-spectrum UV protectant and is thus frequently used in sunscreen preparations. It offers significant photostability and is an approved UV protectant in Europe. It is used under the name TINOSORB^®^ A2B by BASF SE. Methylene bis-benzo triazolyl tetramethyl butylphenol (nano), or MBBT, is another approved UV protectant in the EU market and can be utilized at percentages of up to 10% w/w in dermally applied cosmetic preparations. According to the Scientific Committee on Consumer Safety (SCCS) assessment, MBBT does not represent a danger to people if applied to solid, unbroken skin. Nonetheless, it has raised concerns related to possible harmful impacts and has the potential to bioaccumulate in selected tissues [16].

### 2.3. Bucky Balls (Buckminsterfullerene/C60)

Carbon fullerene has been extensively used in cosmetics and cosmeceuticals due to its antioxidative properties. Fullerenes are widely used in skin-rejuvenating cosmeceutical formulations because of their potent scavenging ability of free radical oxygen species, thus helping to reduce the effects of UV damage, such as hyperpigmentation and wrinkles [51]. Fullerene is a three-dimensional spherical compound that comprises a carbon ring with an odd number of carbon atoms [52] and is hence called “buckyballs” or buckminsterfullerene. Fullerenes alone have limited applications because of their hydrophobic nature, but the use of surface-active agents in a suitable concentration has improved their aqueous solubility and hence has successfully increased their utilization in pharmaceutical applications [36,53].

### 2.4. Miscellaneous

Nanoparticles utilized in cosmetics or cosmeceuticals can be comprehensively classified into two categories: biodegradable nanoparticles (made up of lipids, chitosan, etc.) and non-biodegradable nanoparticles (ZnO, silica-based nanoparticles, etc.) [54]. Chitin and its deacetylated derivative chitosan are another class that is of extraordinary interest to the cosmeceutical industry owing to their special organic and mechanical properties [55]. Nanofibrils of chitin are obtained from the shellfish exoskeleton with the removal of protein fractions and carbonate [54]. Chitin nanofibrils in emulsions can organize into a hygroscopic subatomic film that hinders water dissipation and adds to skin hydration [16,56].

## 3. Nano-Drug Delivery Systems Used in Cosmetics

**Figure 3:**
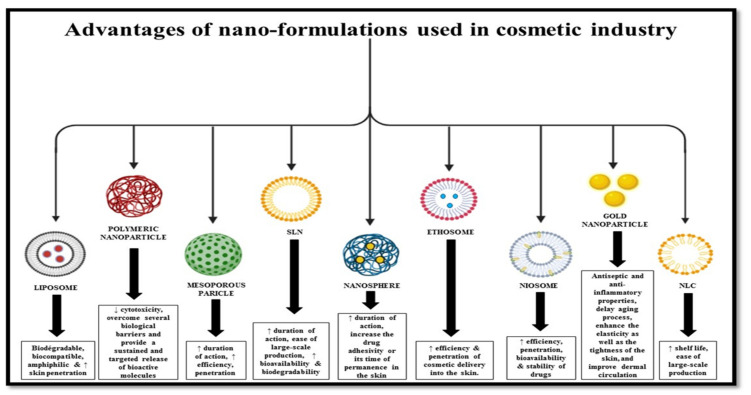
Various nanoformulations used in the cosmetic industry.

Over the past few decades, nanotechnology has been providing novel solutions to several problems in the medical and pharmaceutical arenas. This same concept has been applied in cosmetics, resulting in novel formulations termed nanocosmeceuticals and providing customized remedies for cosmeceutical problems. The novel benefits may be attributed to a smaller size that helps to acquire new properties, such as better solubility, transparency, chemical reactivity, and stability. Several nanomaterials, such as liposomes, ethosomes, solid lipid nanoparticles, nanocapsules, dendrimers, nanocrystals, cubosomes, and nanoemulsions, are used in the cosmetic industry. Currently, cosmetic formulations incorporating nanoscience are extensively marketed. The following sections, including Figure 3 and Table 2, describe various submicron-sized novel drug delivery systems used in cosmetic industries to deliver active ingredients.

### 3.1. Nanoliposomes

These are a nanometric form of liposomes that can be described as vesicles with concentric bilayers, where the fluid volume is encapsulated by bilayers of phospholipids [1], and are widely used as controlled release systems. Conventional liposomes are large and are actually liposomes inside another liposome; hence, they have a limited ability to enter narrow blood vessels or the skin, whereas nanoliposomes have better penetration ability. Being biodegradable and biocompatible, they behave as an exceptionally versatile nanomaterial in the field of cosmetics [68]. Cosmetic formulations incorporating liposomes have greater stability on the skin, as they are not easily washed off. These are ideal carriers of cells and biomembranes and can be successfully applied to the skin because they resemble the biological composition of the skin. They can also be utilized for fixing and transporting nutrients and for imparting pleasant scents to body wash, lipsticks, and antiperspirants [12]. Nanoliposomes in cosmetics enhance the hydration of the skin due to the smaller size of the particles, making the skin smooth and elastic. These are able to transport active moieties into the deeper layers of the skin, even to the systemic circulation, and can act as a transdermal drug delivery system (TDDS) in cosmeceutical applications. However, despite their promising features, low medication stacking, low reproducibility, and physicochemical fragility issues have restricted their commercial applications in beauty care products [16,69,70]. In the cosmetic industry, they are primarily employed for moisturizing and anti-aging purposes.

Recently, Han et al. demonstrated a novel approach to improve the absorption profile of collagen peptides obtained from *Asterias pectinifera* by using elastic nanoliposomes. This combination led to a promising formulation that not only resulted in a reduction in the expression of MMP-1 (produced upon exposure to UV radiation), thus preventing light-induced aging, but also may be used as an eco-friendly source of materials for anti-aging cosmetics [71]. Further, Kocic et al. performed an experiment that compared the moisturizing effect of marketed creams and nanoliposome creams incorporating skimmed donkey milk. They concluded that the nanoliposome encapsulated cream was able to penetrate deeper layers, resulting in reasonable moisturizing capacity with a rapid rate of hydration, and it therefore may contribute to anti-aging activity [72].

### 3.2. Ethosomes

The largest organ of the human body is the skin, which is known to restrict the movement of substances into the systemic circulation owing to the presence of a thick stratum corneum, which acts as a key physiological barrier. Ethosomes containing a very high concentration of ethanol and lipids are soft and flexible vesicles used as carriers to enhance transdermal delivery of a variety of cosmetic agents [73]. They can be customized for the safe and effective skin permeation of cosmeceutical products incorporating antioxidants, anti-wrinkle agents, salicylic acid, and many others. These systems are much more efficient than conventional liposomes in delivering topically applied cosmetics to the skin [74]. 

Research has found that the ethosomal formulation of niacinamide and melatonin can improve their ability to penetrate the skin with increased efficacy [75]. Another study claimed that ethosomes incorporating phenylethyl resorcinol delivered the active agent successfully into the skin for its skin-lightening activity [76,77]. One of the studies carried out by Yücel et al. claimed that the transdermal application of ethosomes loaded with rosmarinic acid (having anti-aging properties) exhibited better efficiency than that of the liposomal formulation. The skin permeation profile for the ethosomal formulation was found to be high with increased transdermal flux as compared to that of the rosmarinic acid solution and liposomes [78]. Another investigation conducted by Pravalika et al. with ethosomal vesicles incorporating minoxidil (a drug for the treatment of baldness) showed that the ethosomal gel had improved penetration as compared to other marketed formulations, which was concluded from both ex vivo permeability and hair growth experiments [79].

### 3.3. Solid Lipid Nanoparticles (SLNs) and Nanostructured Lipid Carriers (NLCs)

These are two novel delivery systems made up of a single layer of shells having a lipoidal center [2,63] and are used for formulating pharmaceutical as well as cosmeceutical products [80]. These formulations are characterized by a solid-state lipid matrix having a size in the nano range. The small size of the formulations permits direct entry into the corneum layer, which enhances the infiltration of active ingredients into the skin [81,82]. They show improved biocompatibility and safety and act as a successful transporter delivery system in cosmeceutical applications [12]. Since 2005, SLNs have been used in several dermal cosmetic products to achieve good outcomes [83,84]. SLNs are widely used in formulating sunscreens, where they act as active carriers for molecular sunscreen agents. They reduce the necessary amount of the sunscreen agent while offering the same protection as compared to conventionally used formulations. SLNs formulated with tocopherol acetate prevented chemical degradation, and improvement in the UV-blocking capacity was reported [85]. Another investigation incorporating a combination of chemical UV absorbers, chitin, and tocopherol in SLNs showed enhanced UVB protection action [86]. They are also used for film formation, which helps in the re-enforcement and repair of the skin barrier, making them perfect for cosmeceuticals that are used to treat irritated and itchy skin and dermatitis. Comparing the two, the crystalline nature of SLNs leads to less drug encapsulation efficiency as compared to NLCs, which have comparatively better encapsulation. Additionally, SLNs have a short shelf life along with slower drug release rates compared to NLCs [83].

A recent experimental study carried out on an SLN formulation integrating fucoxanthin (protects against UVB light) concluded that the presence of the SLN carrier improved the bioavailability of fucoxanthin and can be a promising carrier for sunscreen cosmetics, showing greater stability and good sunscreen-boosting action [84,87]. Another study combined the capability of the flavonoid as a natural antioxidant with NLCs to form an effective system for delivery into the cells. Further, the produced NLCs were incorporated into the skin with good stability and no significant cytotoxicity, suggesting that they can be used as anti-aging and moisturizing cosmetics in the future [88]. 

### 3.4. Nanocapsules

These are polymeric nanomaterials that encapsulate an oily or water phase within them. They are employed in beauty care products for protecting ingredients, masking undesirable odors, and mitigating incompatibility issues between various components in the formulations. Polymeric nanocapsule suspensions can be applied on the skin directly or can be fused into semisolid systems and used as carriers. The level of skin penetration can be regulated by the use of polymers and surfactants in the formulation [89]. In one study, nanoprecipitation was used to fabricate stable poly-l-lactic acid nanocapsules with a size of around 115 nm, and the continual release of fragrance was successfully established by encapsulating odorous atoms in a polymeric nano-transporter [90]. This sort of encapsulation of atoms in biocompatible nanocapsules can assume a critical role in antiperspirant formulations to enhance their effectiveness [16]. Recently, researchers developed novel stimuli-responsive nanocapsules that were developed to carry vitamins and extracts and finally were incorporated into semisolid formulations such as creams. When these formulations were applied on the skin, stimuli induced by damaged skin, such as a pH change and the presence of enzymes, forced the nanocapsules to release their active ingredients at the particular location of the skin [91].

Recently, one study demonstrated the successful incorporation of perfluorodecalin (oxygen carrier) into a silica nanocapsule core as a new tactic for topical therapy of aging skin due to the inherent instability of perfluorocarbon emulsions. Furthermore, this combination displayed better delivery and stability compared to emulsions [92]. Barbosa et al. developed nanocapsules composed of poly(ε-caprolactone) carrot oil and Pluronic containing benzophenone-3 in a sunscreen formulation. These nanocapsules improved the stability of the benzophenone in the topical formulation and also showed synergistic SPF activity with a non-irritant profile [93].

### 3.5. Dendrimers

Dendrimers are three-dimensional nanostructured macromolecules that are extensively branched, and this assembly accounts for their great adaptability [12]. They are generally polymers, and because of their stability, they are helpful in delivering active ingredients through the skin [82]. These molecules can be used in formulating shampoos and antiperspirants with increased efficiency. The surface movement and branches of dendrimers are due to the hydrophobic properties of their peripheral regions combined with the hydrophilic attributes of their central regions [68]. Moreover, properties such as monodispersion, polyvalence, and dependability make them ideal transporters for drug and cosmetic delivery [2]. Dendrimers of resveratrol (having antioxidant and anti-aging activities) have been developed and have assisted in improving the general solubility and skin infiltration [94], which later encouraged the scale-up and commercialization of this dendrimer structure-based formulation [16].

### 3.6. Nanocrystals

These are clusters made up of thousands of molecules joined together in a fixed pattern to form a group with sizes ranging from 10 to 400 nm and are usually utilized for the administration of poorly soluble drugs [95]. Nanocrystals mainly incorporate bioactive compounds and help to improve their dissolution rate. “Juvedical”, developed by Juvena in the year 2000, was the first marketed formulation containing nanocrystals with rutin as a key ingredient [96]. 

A study claimed that nanocrystals of rutin showed higher bioactivity as compared to the normal rutin glycoside [97]. In one of the latest studies carried out by Köpke et al. on the anti-pollution agent SymUrban, the solubility and the penetration profiles were observed to remarkably increase in its nanocrystal form. These nanocrystals increased the dermal bioavailability of the poorly soluble active ingredient in SymUrban and appeared to be a favorable delivery system for this material [98].

### 3.7. Cubosomes

Cubosomes are nanoparticles, particularly fluid crystalline particles, of a specific surfactant with an appropriate proportion of water combined in a nanostructure. Monoglyceride glycerol monoolein is the most common surfactant used to make cubosomes. These are distinct nanostructured particles that are used as cosmeceuticals for skincare formulations and also used in antiperspirant preparations. A number of investigations in collaboration with cosmetic organizations are attempting to utilize cubosomes for absorbing pollutants from cosmeceutical formulations and also employ them as a stabilizer for the oil-in-water type of emulsions [36,99,100].

Khan et al. reported a cubosome formulation containing erythromycin and concluded that the said non-invasive formulation exhibited better activity and effectiveness in preventing and treating acne and worked in a prolonged-release manner [101]. Further, one of the clinical studies conducted by El-Komy et al. claimed that the prepared cubosomal topical gel formulation incorporating alpha-lipoic acid is a safe and efficacious alternative for improving skin aging problems [102].

### 3.8. Nanoemulsions

Nanoemulsions are normally water-in-oil (w/o) or oil-in-water (o/w) colloidal solutions that range from a couple of nanometers to 200 nm [103]. The small size of the droplets is responsible for their alluring optical, rheological, and improved drug delivery properties, as compared to traditional formulations. Further, low viscosity, high solubilization ability, and increased kinetic stability due to sedimentation and flocculation make it more popular. Generally, these are transparent and stable and are employed for cleansing purposes, specifically in the cosmetic industry. These materials are used as powerful vehicles in the cosmetic industry for formulating body lotions, skin creams, sunscreens, etc. Nanoemulsions are also used in designing novel delivery systems for drugs and fatty materials such as essential oils, fatty acids, flavors, and colors. These systems are most appropriate for delivering lipophilic compounds, thus increasing their concentration in the skin; hence, they play a significant role in cosmetic formulations. An increase in patent-filing activity identified for nanoemulsions shows the emerging interest of industries in nanoemulsions [104]. An O/W nanoemulsion incorporating hydroglycolic extract of *Opuntia ficus-indica* (L.) Mill was formulated and characterized by high strength and saturating capacity [105]. Further studies revealed that nanoemulsions could generally impact the distribution profiles of atoms, specifically O/W nanoemulsions, which significantly improved the penetration profiles of polar ingredients relative to traditional emulsions [16,106].

Antioxidants play a significant role in the cosmetic and pharmaceutical industries but suffer from various limitations, such as insolubility and instability. To overcome these problems, researchers have fabricated antioxidants as nanoemulsions with enhanced efficacy [107]. In one of the investigations, to overcome the poor aqueous solubility problems of ellagic acid, Zhang et al. fabricated an oil-in-water nanoemulsion. They concluded that the developed nanoformulation had increased aqueous solubility and permeability through the skin, thus strengthening its whitening effect [108].

### 3.9. Micellar Nanoparticles 

These are recognized as one of the most effective nanotechnology-based particles and have been widely used in the cosmetic industry. They offer a robust and versatile platform to encompass wide-ranging lipophilic active ingredients possessing diverse physicochemical properties in cosmetic formulations. Smaller particle size, better encapsulation efficiency, and reasonable manufacturing cost are the key features of these particles that make them more efficient than other nanocarriers [109,110]. Usually, they are employed in skin cleansing products for effective removal of oil and dirt from the skin without affecting barrier integrity and are used as an alternative to conventional cleansers [36,111]. These nanoparticles have led to a revolution in transdermal drug delivery (TDD). Micellar nanoparticle-based emulsions are attractive candidates for systemic drug delivery through topical application. The technology permits a high concentration of the drug to permeate the skin, creating a drug formulation attaining the same benefits as those of TDD, making the formulation more acceptable. Facial cleansing formulations incorporating micellar nanotechnology are claimed to be the most effective products by different cosmetic brands. 

In one of the investigations by Zięba et al., the authors formulated a micellar shampoo and concluded that it had a higher viscosity and increased ability to emulsify fatty deposits as compared to traditional shampoos. 

## 4. Health Risks Associated with Nanocosmeceuticals

It has been well established that nanoparticles pose serious health risks to humans due to their potential toxicity, which may further depend upon the quantity, route, and time of exposure of the nanoparticles. Other factors may include shape, surface structure, surface charge, chemical composition, and solubility [112]. Due to their small size and shape, nanoparticles can move easily inside the human body and are able to cross membranes and gain access to cells, tissues, and organs that are not accessible to larger-sized particles [113]. They can even enter cells, causing more damage or cell death [114].

At the nanoscale, the fundamental properties of substances become altered. For the same substance, the physicochemical properties differ between the nanoparticulate and larger particulate states. At the nano level, the chemical reactivity and biological activity are often higher as compared to those of larger-sized particles, which is attributed to their higher surface-area-to-volume ratios. Further, nanoparticles experience higher chemical reactivity, resulting in increased production of reactive oxygen species (ROS), including free radicals [115]. This is one of the key mechanisms of toxicity initiation that may lead to inflammation, oxidative stress, and consequent damage to membranes, proteins, and DNA. Nanomaterials may induce toxicity in various human systems, such as pulmonary, reticuloendothelial, neurological, and cardiovascular systems, and also have endocrine-disrupting or immunological effects.

The entry of nanoparticles into the body primarily occurs by three different routes, namely, inhalation, ingestion, and through the skin.

Inhalation is the most widely recognized route of exposure to airborne nanoparticles, as per the National Institute of Occupational Health and Safety (NIOHS). For instance, during the manufacturing of nanocosmeceutical products, workers may be exposed and may breathe in nanomaterials. Further, customers may also breathe in nanomaterials when applying products that contain them. For example, sunscreen sprays consisting of nanoscale TiO_2_ may cause the inhalation of nanomaterials, which may travel through the nasal nerves to reach the cerebrum (brain) and sensory system and enter the blood and various organs, causing life-threatening adverse effects [116,117]. 

Further, the ingestion of nanomaterials may take place accidentally via transfer from the hand to the mouth or may be ingested intentionally. After ingestion, a moderate amount of the nanoparticles may be taken up by the body and move into vital organs and tissues, causing side effects [116,117].

**Figure 4:**
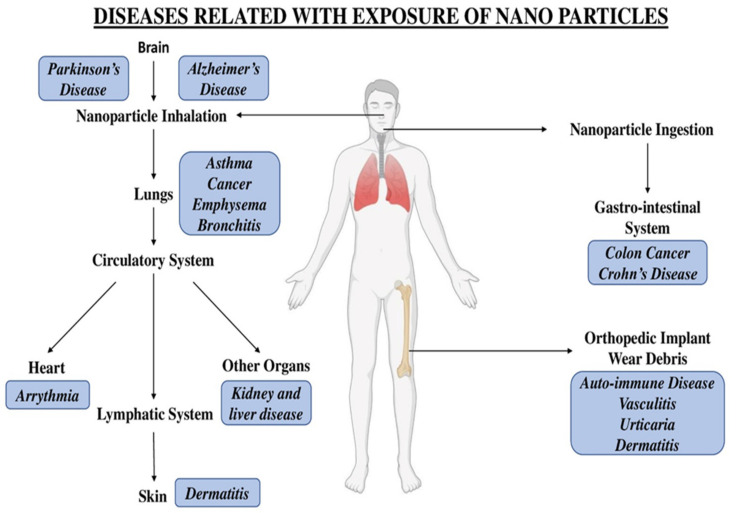
Diseases associated with nanoparticle exposure [119].

Topical use of nanomaterials may also cause harmful effects. Various experimental data have shown that certain nanomaterials gain entry into the deeper layers of pig skin within 24 h of exposure [118]. As per investigations by the US Government Accountability Office (GAO), nanomaterials present in sunscreens can enter through damaged skin, causing serious side effects [116,117]. Figure 4 illustrates different diseases related to nanoparticle exposure.

As per the Yearly Meeting of the American Association for Disease Research, 2007, nanoparticles can damage DNA and lead to malignancy. Nanoparticles are small enough to infiltrate cell layers, yet they are large enough to alter normal cell functions, according to researchers at the University of Massachusetts. Because of their tiny size, they can be hard to separate by traditional separation procedures, and they can come across malignant cells and can cause significant damage, as per Sara Pacheco, a scientist at the College of Massachusetts. However, due to their small size, it is difficult to confirm exactly how they interact with the environment and how they affect human health [120]. 

Neonatal toxicity is another area of toxicity where exposure to these particles may cause severe harm in pregnant women. Nanoparticles used for various purposes may enter the placenta, endometrium, yolk sac, or fetus, resulting in oxidative stress and irritation. These problems may lead to placental damage, delayed neonatal development, fetal deformities, neurotoxicity, and reproductive dysfunction in infants. Further, nanoparticles may induce cytokine production in pregnant women, which may enter the fetus and result in poor development of the fetal brain [121].

## 5. Environmental Risks of Nanoparticles

In the modern era, nanotechnology is exploited as a useful tool to improve the quality of the environment, such as water and air, by cutting down waste production, reducing the emission of greenhouse gases, and decreasing the discharge of hazardous chemicals in the environment. However, they also have a negative impact on the environment. Owing to the novel and unique physical and chemical properties of nanoparticles, they can easily enter very small spaces [122,123]. They can cause biochemical interference by participating in reactions in biological systems. The mechanisms by which a nanomaterial can cause cell impairments can include bioaccumulation, ROS formation, oxidative stress, autophagy or lysosomal dysfunctions, etc. [124]. The influences of nanoparticles on the environment depend on the way that they are used in the workplace, their segregation mechanism in diverse media (such as air, water, and soil), their mobility, and their stability. Further, nanotechnology exposure in the environment and transportation are the fundamental factors that determine the overall influence on the environment. Nanoparticles are essential elements in various biogeochemical processes, and hence, any global-scale impact of specific nanoparticles on elemental cycles should be considered. Additionally, the environmental impact of these particles depends on factors such as solution chemistry, biochemical reactions, redox potential, temperature, pressure, presence of coating, etc., which should also be taken into consideration. 

During the manufacturing process, nanomaterials may discharge into the water, air, and soil, causing serious environmental risks. As indicated by an investigation by the US Government Accountability Office (GAO), nanomaterials having antibacterial properties, whenever delivered in sufficient quantities, might interfere with the useful activity of microbes in water treatment plants and result in failure of the water proposed for reuse [1]. Nanoparticulate TiO_2_, used to decompose pollutants and for disinfection purposes, may have the ability to stimulate other organic transformations and have an influence on photochemical reactions in the atmosphere. Likewise, in an investigation coordinated by the University of Toledo, it was found that nano-TiO_2_, which is considered a close-to-home element, reduced the organic functions of microbes after an hour of exposure. Hence, it has been suggested that these particles, which end up at civil sewage treatment plants, could affect organisms that provide indispensable functions in the environment and thus should be used judiciously [1].

It has been evident that nanoparticulate TiO_2_, which is a key constituent of sunscreen, is released in significant quantities, causing potential damage to marine life with chronic exposure. In many sunscreen products, TiO_2_ acts as a protective chemical coating, but when exposed to water, this defensive coating tends to be lost due to the influence of either UV light or seawater composition, releasing the toxic TiO_2_ into the aquatic environment, specifically to algae and daphnids. This ultimately affects aquatic ecological balances [125,126]. Carbon-based nanomaterials show severe cytotoxic effects not only in human beings but also in other mammals by accumulating in different organs, such as lungs and kidney tissues [127]. At higher concentrations, they disturb the metabolic activity of microbes by interrupting the biogeochemical cycle of nutrients and also upsetting the nutrient balance. Further, metal nanoparticles have a high affinity for proteins [128] and can induce harmful cellular reactions and produce toxic effects on cells. An investigation carried out on the effects of titanium, polystyrene, and fullerene nanoparticles indicated that they are more toxic under biotic conditions than abiotic conditions because they induce oxidative stress [129].

In one of the studies carried out on carbon fullerenes, it was concluded that they can cause mild harm in largemouth bass (fish) [130], an animal used as a model for characterizing ecotoxicological impacts. Fullerenes have similarly been found to destroy water fleas and have bactericidal properties [131]. The Center for Biological and Environmental Nanotechnology of Rice University, Houston (USA), has called attention to the binding of nanoparticles to chemicals in the climate, such as cadmium and petrochemicals. This tendency would make nanoparticles a likely component of long-term and long-range vehicles of contaminants in groundwater [132,133].

These nanomaterials also affect plants after their absorption and translocation, producing severe effects. Nanoparticles of Ag, ZnO, fullerenes, silica, etc., are taken up by plants and algae, thereby producing toxicity and also hindering seed germination, as confirmed by researchers. They also impede the metabolic pathways affecting the growth and functioning of cells. In this way, these particles may enter the food chain and become biomagnified.

A workshop conducted by the National Science Foundation and the US Environmental Protection Agency was launched to identify the critical risk issues relating to nanomaterials. The workshop’s specific goals were to determine the exposure and toxicity of manufactured nanoparticles, the ability to deduce manufactured nanoparticle toxicity using existing particle and fiber toxicology archives, and the recyclability and total sustainability of manufactured nanomaterials.

At present, there is very little information available relating to the environmental risk of manufactured nanomaterials. Only a few findings have been published that describe the direct and indirect impacts of nanomaterials. To assess their risks, it is essential to have basic information about the behaviors and risks of nanoparticles. However, to date, there are no accurate data or guidelines available to quantify such effects. Further, it is essential to identify the sources, environmental paths, and uses of nanoparticles, along with plants and animals that are sensitive to nanoparticles, for the environmental risk assessment.

Currently, several researchers are determining the concentrations of a variety of nanoparticles in indoor and outdoor working areas. This helps to determine the levels of exposure of employees for that particular purpose and also for precautionary measures. Few studies have been published on the impacts of this type of exposure to nanomaterials. However, extensive research is still needed to evaluate the interactions of nanoparticles with biological systems as well as the ecosystem. Additionally, research should be conducted to establish the absorption, interaction, biodistribution, and excretion pathways of nanoparticles in living systems, along with nanotoxicological studies [134].

The biosynthesis of new nanomaterials from existing ones, polymeric coatings of metal nanoparticles to prevent leaching, combustion of carbon-based nanomaterials, recycling of metal-based nanoparticles, and bioaccumulation by means of plants or fungi can be used as effective methods for removing nanoparticles from different media. Green technology or green manufacturing can be used as a remedial solution for ecological protection. This is an environmentally friendly technology that intends to produce nanoparticles with reduced raw materials, minimum energy utilization, and less waste production, aiming to conserve natural resources. It employs green chemicals that are less toxic to the environment and is an energy-efficient procedure [135,136]. Lastly, awareness of individuals about the toxicity of nanomaterials, as well as attention to the use of safe and eco-friendly alternatives, may help resolve the problem [137].

## 6. Regulatory Guidelines of Cosmetics and Cosmeceuticals

The worldwide cosmetic market, valued at USD 532.43 billion in the year 2017, is predicted to reach USD 805.61 billion by the year 2023, with an annual growth rate of 7.14% [111]. Table 3 lists various patents linked with cosmeceuticals that may highlight the current global trend of cosmeceuticals. Looking at this increasing market value, it is necessary to regulate the cosmetics market with appropriate legal guidelines that will help to ensure the well-being of consumers. Although it is challenging to fulfill legal obligations for personalized products, it can be achieved with the correct approach and sustained compliance.

There are many regulatory documents that are intended to assist the cosmetics industry and other stakeholders (academicians, researchers, etc.) in identifying and investigating the safety aspects of nanomaterials in cosmetics. There are compilations in the literature that summarize the legal aspects of nanomaterials [138] or the use of nanomaterials specifically in cosmetics [139]. Here, we briefly summarize the main recommendations of a few important guidance documents.

### 6.1. Food and Drug Administration (FDA): Guidance for Industry Safety of Nanomaterials in Cosmetic Products

This document provides guidance to industry and other stakeholders on the FDA’s current thinking on the safety assessment of nanomaterials in cosmetic products. The FDA’s guidance documents, including this guidance, do not establish legally enforceable responsibilities. Instead, guidance should be viewed only as recommendations unless specific regulatory or statutory requirements are cited.

This guidance also refers to other relevant reports, such as the Organization for Economic Co-operation and Development (OECD) Working Party on Manufactured Nanomaterials “Preliminary Review of OECD Test Guidelines for their Applicability to Manufactured Nanomaterials”, the Scientific Committee on Consumer Safety (SCCS) “Guidance on the Safety Assessment of Nanomaterials in Cosmetics”, and relevant ICCR reports, such as on the “Currently Available Methods for Characterization of Nanomaterials,” and “Principles of Cosmetic Product Safety Assessment.”

This guidance presents recommendations very comprehensively. However, in summary, for any cosmetic product that has new or altered properties, data needs and testing methods should be evaluated to address any unique properties and functions of the nanomaterials used in the cosmetic products. The FDA recommends that the safety assessment of cosmetic products using nanomaterials address several important factors, including:

- The physicochemical characteristics,
- Agglomeration and size distribution of nanomaterials under the conditions of toxicity testing and as expected in the final product,
- Impurities,
- Potential routes of exposure to the nanomaterials,
- Potential for aggregation and agglomeration of nanoparticles in the final product,
- Dosimetry for in vitro and in vivo toxicology studies, and
- In vitro and in vivo toxicological data on nanomaterial ingredients and their impurities, dermal penetration, potential inhalation, irritation (skin and eye), sensitization studies, and mutagenicity/genotoxicity studies.

The safety of a cosmetic product should be evaluated by analyzing the physicochemical properties and the relevant toxicological endpoints of each ingredient in relation to the expected exposure resulting from the intended use of the finished product. If the manufacturer wishes to use a nanomaterial in a cosmetic product, either new material or an altered version of an already marketed ingredient, this guidance recommends that it meets with the FDA to discuss the test methods and data needed to substantiate the product’s safety, including short-term toxicity and other long-term toxicity data, as appropriate [140].

### 6.2. International Cooperation on Cosmetics Regulation (ICCR): Report of the ICCR Working Group—Safety Approaches to Nanomaterials in Cosmetics

Discussions at the 4th annual meeting of International Cooperation on Cosmetics Regulation (ICCR-4) on cosmetics and cosmetic-like drugs in Canada in July 2010 led to the formation of a Joint Industry/Regulator Working Group (WG) for nanomaterial safety. The purpose of this Joint WG was to examine existing safety approaches for their applicability to nanomaterials relevant to activities within the cosmetic industry. The main task was to carry out a review of existing safety approaches, identify any specific aspects relevant to consumer safety that should be taken into consideration when assessing nanomaterials in cosmetics, and produce a draft report for discussion by the ICCR members.

The members of the Joint WG discussed the main issues and prepared a report after considering several key reports, opinions, guidance documents, and relevant publications. The report’s aim is to provide information to those intending to use or assess the safety of nanomaterials in a cosmetic product. This report expresses the views of the experts on the key safety aspects that need to be assessed when using nanomaterials in cosmetic products.

The main highlights of the report are:

- The existing risk assessment pattern (exposure assessment, hazard identification, hazard characterization, and risk characterization) used for conventional chemicals is also applicable to nanomaterials.
- The WG identified physicochemical parameters that should be measured for nanomaterials at the raw material stage.
- The assessment should include the investigation of systemic exposure, local effects, possible routes of exposure (dermal, respiratory, or oral), and foreseeable uses of the cosmetic product.
- If systemic absorption is seen, it should be further investigated to confirm whether the absorbed material was in particle form or in a solubilized/metabolized form. The absorption, distribution, metabolism, and excretion (ADME) profile should be investigated to assess the fate and behavior of the nanoparticles in the body and identify the plausible target organs.
- For nanomaterials having very low absorption, processes such as accumulation should also be considered.
- The effects of the formulation should also be considered, as certain formulations may alter the bioavailability and toxicological profile of active ingredients.
- The EU Cosmetics Regulation bans the testing of cosmetics on animals. This makes the safety evaluation of new nanomaterial cosmetic ingredients difficult. Though there are several validated alternative methods that can be used in place of animal tests for conventional substances, none of those methods is yet validated for nanomaterials. However, they may still be useful for hazard identification and provide additional supporting evidence to the results of in vivo studies [141].

### 6.3. Scientific Committee on Consumer Safety (SCCS): Guidance on the Safety Assessment of Nanomaterials in Cosmetics

This document is an up-to-date guidance on the safety assessment of nanomaterials in cosmetic products, covering the main elements of safety assessment, i.e., general considerations, material characterization, exposure assessment, hazard identification/dose–response characterization, and risk assessment. 

The main points of this guidance may be summarized as follows.

#### 6.3.1. Definition of Nanomaterial

Material specifications such as particle size distribution, solubility, and persistence should be considered to decide whether a cosmetic ingredient is a nanomaterial or not. Imaging by electron microscopy may be used for further clarification if needed. Where a cosmetic ingredient qualifies to be defined as a nanomaterial, it will be subjected to safety assessment based on the data relevant to nanoscale properties.

#### 6.3.2. Material Characterization

Considering the special behavior of the nanomaterials, their clear identification and detailed characterization are essential requirements for safety assessment. The characterization of the nanomaterial needs to be carried out at the raw material stage, in the cosmetic formulation, and during exposure for toxicological evaluations. The characterization data must identify the materials in accordance with Cosmetics Regulation (EC) No 1223/2009. Measurements must be carried out using generally accepted techniques, and detailed documentation must be provided. Particle size, being the most important factor, must be measured by more than one method, one of which should be electron microscopy.

#### 6.3.3. Exposure Assessment

Safety assessment of nanomaterials is performed using the same procedure as for non-nano-ingredients, but with special attention to the nano aspects. Firstly, the likelihood and extent of local and systemic exposure need to be determined in relation to dermal, oral, and inhalation exposure routes. The potential translocation of nanoparticles across skin, lung, or gastrointestinal barriers should be determined. The methods used for this purpose should be mainstream and state-of-the-art with a low limit of detection. ADME parameters should be studied to determine the extent of systemic exposure, fate, and behavior of the nanomaterial and to identify the target organs. If systemic exposure is indicated, further investigations should be carried out to confirm whether the absorbed material was in particle form or in a solubilized/metabolized form. In cases where systemic exposure is not indicated, local exposure and local adverse effects should be investigated.

#### 6.3.4. Hazard Identification/Dose–Response Characterization

Data from toxicological studies on local/systemic effects are required as per SCCS Notes of Guidance. Hazard identification/dose–response characterization includes consideration of insoluble or partially soluble particulate forms, aggregation and agglomeration behavior of the particles, potential penetration of nanoparticles through biological membranes, possible interaction with biological entities at local and systemic levels, surface adsorption/binding of other substances, surface-catalyzed reactions, persistence, etc. The prohibition of animal testing as per the Cosmetics Regulation must be observed in any toxicological testing. The SCCS can accept results from methods that may not have been formally validated for nanomaterials but can be demonstrated to be scientifically valid for hazard identification, provided that they are carried out with due consideration of the nano-related aspects and appropriate controls.

#### 6.3.5. Safety Assessment

With the EU ban on animal testing of cosmetic ingredients/products, the applicant needs to collect relevant data from different alternative methods and provide evidence to support the safety of the cosmetic ingredient. Where safety assessment is based on in vitro test results, extrapolation of in vitro to in vivo (IVIVE) data will be required [142].

### 6.4. Comparison of Regulation of Cosmetics/Cosmeceuticals across Different Countries 

The safety and efficacy of cosmetic products are governed by different regulatory bodies of countries all around the world according to their own guidelines. In a few countries, the final product’s safety is assured before marketing approval by the manufacturers. The label should contain all constituents of the formulation along with the limits that are identified for the cosmetic and cosmeceutical ingredients and products, and the mentioned limits should comply with the established limits. Simultaneously, many countries lack these regulations. The following section lists a few of the current regulatory scenarios of cosmetic products in the United States of America (USA), the European Union (EU), and India [7], and Table 4 compares the regulation of cosmetics/cosmeceuticals in these regions. This may help the reader to understand various regulatory procedures in different countries.

#### 6.4.1. United States of America

In the USA, the regulation of cosmetics is approved by the United States Food and Drug Administration (USFDA) and controlled by the Food Drug and Cosmetic Act (FDCA). It is well known that drugs are regulated by the FDA. They must either have FDA’s premarket approval or follow the final regulations specifying conditions to be recognized as safe and effective, but cosmetics lack this premarket approval procedure, except for color additives [7].

The most commonly used cosmetics include toothpaste; nail polish, skin, eye, and facial creams, lotions, lipsticks, perfumes, antiperspirants, shampoos, hair products, etc. Soaps mainly comprising a soluble base salt of unsaturated fat used for cleaning the human body are not regarded as cosmetics as per the law (USFDA, 2014). Cosmetic products do not have comprehensive rules for approval before their marketing, in contrast to drugs. In the United States, at a minimum, the manufacturers, distributors, and packagers of the cosmetic item are expected to use the Voluntary Cosmetic Registration Program (VCRP), which offers benefits for participation. VCRP provides information to the FDA about beauty care products and their manufacturing, distribution, and recurrence activity. The producer or wholesaler must prepare a documentary report, which is known as a Cosmetic Product Ingredient Statement, for each item that the firm has brought to the market. According to the law of the organization, the USFDA might carry out an examination, inspect items and the organization wherein items are manufactured or stored, and identify misbranded or tainted cosmetic or cosmeceutical products. The Personal Product Act 2013 was established to enable the USFDA to guarantee that cosmetic items are completely safe and contain no harmful entities [7].

The cosmetic regulatory guidelines that apply to contaminated and misbranded products are provided in the Federal Food, Drug, and Cosmetic Act, which requires a one-year-long registration of an operation involving advertisement, manufacturing, or dispensing cosmetics. It also requires the disclosure of data and the labeling of active ingredients as well as excipients and also should reveal the related adverse effects, if any [7].

The act requires the organization to maintain standard records of restricted constituents and the constituents that are completely safe and unregulated for the purpose of utilization in cosmetic formulations. Along with this, the manufacturers are required to conduct certain basic important tests of the constituents to ensure their safety. Additionally, this act puts forth prerequisites identified for nanotechnology in the production of cosmetics, compulsory and voluntary review of beauty care products, and alternatives to testing on animals. To share the information, the act establishes the Interagency Council on Cosmetic Safety (ICCS) and, just as importantly, helps the organization that looks after the safety of cosmetics with government analysts. As per the Federal Food, Drug, and Cosmetic Act, 2013, cosmetic products that fail to justify their labels are considered misbranded. The label and the packaging of the product should provide buyers with exact information on how to use or apply it, and the packaging should have information on the comparable quantities of ingredients in the particular product as per the Fair Packaging and Labeling Act. Additionally, the above-mentioned label should be wrapped around the product or placed within it. The principal display panel (part of the label most plainly visible when displayed under the standard environmental factors in settings where it is available for purchase) should show the item name and provide an accurate report of the net quantity of ingredients in the formulation as a measure, weight, count, or a combination thereof. The declaration should be noticeable, situated on the front of the packaging, and at a size proportionate to the size of the package. The packaging must incorporate inserts, booklets, risers, or some other printed or data associated with the product (Cosmetic Labeling Guide, 2015). All of the essential guidelines should be written in the English language and should be placed within the label in such a way that they can be easily noticed and observed by the consumer [7].

In 2006, the FDA established an interior nanotechnology team to manage nanoparticle-based items. This step was taken to improve the safety and effectiveness of nanomaterials. Later, in 2007, alterations were recommended by the FDA; many have been executed, and a few are under investigation. Further, in 2014, the FDA identified three rules concerning the safety of nanoparticles; two of them are associated with cosmetic products. The first rule elucidated the assurance of FDA-managed formulations incorporating nanoparticles. The second one is focused on the safety of nanomaterials in cosmetic items. Additionally, the FDA has not been required to disclose the list of nanomaterials incorporated in formulations on labels [143] and regularly updates manufacturers about nanomaterial-linked risks for continual improvement in the safety of the cosmetics. By implementing this process, formulations are continuously adjusted so that the utilization of hazardous substances is limited [111].

#### 6.4.2. European Union

The European Medicines Evaluation Agency (EMEA) is the regulatory agency for cosmetics in Europe, which is under the direct control of Council Directive 76/768/EEC. It covers the safety associated with the use of cosmetic items and the record of permitted colorants. The safety of cosmetics and smooth operation for all administrators in this area are governed by European Regulation 1223/2009. The above-mentioned regulation provides a strong, universally recognized system that establishes product safety considering the most recent scientific data, including the feasible utilization of nanomaterials [7].

According to the EU, a product safety report should be made prior to entering the market. Only cosmetic products for which a natural or legal individual is delegated can be sold on the cosmetics market; genuine undesirable effects should be communicated to public government agencies, which will later accumulate related data from health experts and clients and will inform the other EU Member States. Preservatives, colorants, and UV protectants with materials in the nano range should be approved. Formulations containing nanomaterials are not under the control of the EU cosmetic regulation and have to go through a complete examination under the supervision of EU experts. The manufacturer will provide information about its formulation with the help of an EU notification portal known as the EU cosmetic products notification portal (CPNP); it should mention the market name and the registered address of the manufacturer [7].

European Commission Regulation No. 1907/2006 regulates nanomaterials in the EU. The nanomaterial ingredients should be suffixed with the term “nano”, e.g., “zinc oxide (nano)”, as per European Commission, 2015 [144]. According to EU guidelines, nanomaterials are characterized as insoluble material and deliberately fabricated with at least one external measurement or an inner dimension in the size of 1–100 nm. Data on the item detailing undesirable effects, the safety profile, and toxicity should be provided half a year before the market approval of nanocosmeceutical/nanoparticle-based items. It requires premarket approval for nano-based cosmeceuticals, anti-aging creams, colorants, and sunscreen items [111]. 

#### 6.4.3. India

In India, the cosmetic market is known to be the quickest rising retail segment, and the active Indian market offers freedom for foreign brands. It permits access to imported beautifying agents with no restrictions. In the last 20 years, many different participants have entered the Indian cosmetic market, hence demanding strict regulations to preserve the safety of consumers. The Central Drug Standard Control Organization (CDSCO) regulates all activities related to cosmetics or cosmeceuticals in India and is controlled by the Drug and Cosmetics Act and Rules. Further, the Bureau of Indian Standards (BIS) regulates the labeling contents of cosmetics or cosmeceuticals. It sets the minimum quality of cosmetic products for the recorded items and provides details about hair care products and creams. Under CDSCO, the Drug Controller General of India (DCGI) regulates all related activities [7]. 

In India, as indicated by the Drug and Cosmetics Act, labels, whether external or internal, should contain the name of the cosmetics and the address of the manufacturer. If the size of the package is small, then the name of the manufacturer’s address with a pin code is sufficient. The external label should contain the name of the ingredients along with their quantities in the formulation. The internal label should include directions for the use of the product and the name and quantity of poisonous or hazardous substances that are used, along with warnings, if any. The particular batch number, which is indicated by the letter “B”, should be included in all cosmetic or cosmeceutical formulations, but in the case of soap, the manufacturing month and year must be present, omitting the letter “B” in the label. However, this is not the case for solid or semisolid cosmetic formulations having a weight equal to or less than 10 g and for liquid cosmetic formulations having a volume equal to or less than 25 mL. The manufacturing license number must be present on the label, which is indicated by the letter “M”, as per Drugs and Cosmetics Act and Rules, 2013 [7].

The Government of India invested in the nanoscience and technology initiative and provided efficient resources to different colleges, scholar societies, public research facilities, and new companies with R&D units. In India, the important bodies involved in the public health research frameworks are the Indian Council of Medical Research, the Department of Science and Technology, the Council of Scientific and Industrial Exploration, and the Department of Biotechnology (all in New Delhi, India). The Ministry of Health and Family Welfare (New Delhi, India) plays an essential role in the prevention and control of health-related issues in India. Furthermore, the Nanotechnology Sectional Committee, which includes specialists subsidiary to different research foundations and associations, is effectively responsible for the standardization of nano-based items and their safety [111]. Currently, nanomaterial concerns are continuously evolving in India and demand special attention for the improved safety of the public. 

## 7. Conclusions and Future Direction

Currently, nanotechnology is regarded as a promising and revolutionizing field and is being utilized and appreciated in the areas of cosmetics, cosmeceuticals, dermatology, biomedical applications, etc. The introduction of newer advancements and novel drug delivery systems make cosmetics and cosmeceuticals more popular with increased market share. Today, these cosmetics are an indispensable part of the daily routine; further, the introduction of nanotechnology to cosmetics has enhanced its acceptance among users all around the world. However, its associated toxicity owing to its penetrability is a major concern that is often overlooked, leading to adverse health issues. Presently, novel nanocarriers such as liposomes, ethosomes, cubosomes, NLC, SLNs, nanoemulsions, niosomes, etc., are exploited to formulate various cosmetics and cosmeceuticals with enhanced outcomes. Nanosystems carry and deliver these formulations across the skin by diverse mechanisms and impart several functions, such as sun protection, moisturization, wrinkle reduction, etc. Even though these nanomaterial products are gaining impressive market value, there is tremendous debate concerning their safety and toxicity in humans, demanding more careful investigations. Hence, the cosmetic legislation should provide a specific list of references as well as the ingredients that produce unintended environmental effects for all users of cosmetic products, such as consumers and professional users, thus ensuring the safety of the usage of cosmetic products. Long-term toxic or carcinogenicity studies of cosmetics, including nanocosmetics and nanocosmeceuticals (and their ingredients), should be conducted before the commercialization of these products. Nanocosmeceuticals should be manufactured in such a way that they add value to the health of consumers. Moreover, careful clinical trials of cosmeceuticals should be conducted, such as those performed for drugs, to assure the safety of the formulations in humans. Additionally, stringent regulations should be imposed on the manufacturing, storage, import, and marketing of cosmeceuticals and nanoparticles incorporated therein. Universal collaborative efforts among researchers as well as global regulatory agencies are required to develop standard rules and regulations for using nanosystems in cosmetics and help address the existing gaps in the related data. Non-governmental organizations and government bodies should work in a coordinated manner to develop and propagate effective education materials for consumers. They should establish special programs, such as written and video materials, through multimedia or seminars with the aim to provide education for the wise use of cosmetics containing nanocosmetics and nanocosmeceuticals. Finally, there is a need to harmonize regulations internationally to establish a better regulatory framework for safety, efficacy, and marketing, which ultimately helps the cosmetic industries and also protects consumers from potential hazards. Moreover, awareness among consumers can also help to improve this situation by enabling informed choices of products.

## Figures and Tables

**Table 1:** Different nanomaterials used for preparing cosmetics and cosmeceuticals.

S. No.	Nanomaterial	Advantage	Disadvantage	Uniqueness	Type of Cosmeceutical	Commercially Available Product	Reference
**1.**	Inorganic particles (TiO_2_, ZnO)	Hydrophilic, biocompatible, safe, and stable	Pulmonary toxicity	Absorb/reflect UV light	Sunscreen	Phytorx UV Defense Sun Block SPF 100—Lotus Professionals	[16,17]
**2.**	Silica (SiO_2_)	Hydrophilic, ↓ manufacturing cost	Pulmonary toxicity	Used as filler to ↑ the bulk of the cosmetic formulation	Lipstick	Face FWD >> Blush Stick—Sugar Cosmetics	[16]
**3.**	Carbon black	Light weight, ↑ chemical and thermal stability, and ↓ cost	Cytotoxicity; alters the phagocytic property of macrophages	Color pigment	Facemask	Face Masque—Carbon BAE	[16,18]
					Mascara	Mascara Black—Lakme	
**4.**	Nano-organic (tris-biphenyl triazine)	Powerful and photostable filter	Hazardous to the aquatic environment	Most efficient UVB and UVA 2 filter	Sunscreen	Extra UV Gel—Allie	[16,19]
**5.**	Nano-hydroxyapatite	Dental desensitizer and polish remineralization of teeth	Very brittle nature	Safe in pediatric toothpaste	Toothpaste	Kinder Karex Hydroxyapatite	[16,20,21]
						APAGARD M plus—Sangi	
**6.**	Gold and silver nanoparticles	Uniform shape, size, and branch length; tuned pharmacokinetics and biodistribution; antibacterial and antifungal activity; and chemical stability	Damages human cells and DNA at high doses; pulmonary toxicity	Surface-enhanced Raman scattering	Facemask	Gold Radiance Peel Off Mask–VLCC	[16,21,22,23]
					Anti-aging cream	Nano Gold Firming Treatment—Chantecaille	
**7.**	Buckyballs (buckminsterfullerene/C60)	Exhibits antioxidant activity, thermostability, and photostability; prevents many skin problems related to oxidative stress	Pulmonary toxicity; damages brain tissues; highly hydrophobic	Potent scavenger of free radicals	Face cream	Brightening Essence—Juva Skincare	[24,25,26]

**Table 2:** Nanoformulations used for preparing various cosmeceuticals.

S. No.	Nanoformulation	Advantage	Disadvantage	Uniqueness	Type of Cosmeceutical	Commercially Available Product	Reference
1.	Nanoliposomes	Biodegradable, biocompatible, amphiphilic, and ↑ skin penetration	May trigger an immune response, ↓ medication stacking, ↓ reproducibility, and physicochemical flimsiness	Ability to compartmentalize and solubilize both hydrophilic and lipophilic materials	Moisturizer	Dermosome—Microfluidics	[16,23,57]
					Anti-wrinkle cream	Capture Totale—Dior	
2.	Niosomes	↑ Efficiency, penetration, bioavailability, and stability of drugs	↑ Cost of production, physical and chemical instability, leakage of the drug, time-consuming production	Surface development and alteration are extremely simple due to presence of useful functional groups on the hydrophilic head	Anti-aging cream	Lancome^®^—Loreal, Paris	[58]
3.	Ethosomes	↑ Efficiency and penetration of cosmetic delivery into the skin	Poor yield problems, ↓ stability, and possibility of coalescence	Consist of a relatively high percentage of ethanol	Moisturizer	Supravir Cream—Trima, Israel	[59,60]
4.	Sphingosomes	Reestablishment of barrier function of skin and repair of dehydrated and damaged skin	Poor entrapment efficiency and expensive	Consist of sphingolipid, which makes them more stable than phospholipid liposomes	Anti-cellulite cream	Noicellex—NTT, Israel	[58,61]
5.	Solid lipid nanoparticles (SLNs)	↑ Duration of action, ease of large-scale production, ↑ bioavailability and biodegradability	↓ Shelf life, decreased drug encapsulation	Crystalline in nature, ↑ drug loading matrix; consists of solid lipid	Perfume and cream	Chanel Allur	[16,62,63]
6.	Nanostructured lipid carriers (NLCs)	↑ Shelf life, ease of large-scale production	↓ Duration of action, higher drug encapsulation	The matrix consists of a blend of solid and liquid lipids	Face spa cream	Dr. Rimpler—Cutanova	[16,62]
7.	Nanocapsules	Protection of ingredients, masking of undesirable odors, resolution of incompatibility issues between formulation components, sustained release formulation	Additional purification step is required after nanocapsule formulation	Formation of micelles and amphiphilic in nature	Anti-wrinkle cream	Primordiale Intense—L’Or’ea	[16,21,64]
					Hair care	Nano Collagen—Braziliss	
8.	Dendrimers	↑ Solubility of the lipophilic drug, controlled-release drug formulation, and maintenance of the stability of the drug in cosmetic formulations	Not good materials for hydrophilic drugs, cellular toxicity, ↑ manufacturing cost	↑ Shelf life of the cosmetic formulation	Sunscreen	Topical Resveratrol Formulation	[16]
9.	Nanoemulsions	Transparent, stable, and amphiphilic	Preparation is difficult in cosmetic formulations, acid-sensitive, and ↓ duration of action	Creams containing nanoemulsions do not show problems of inherent creaming, flocculation, coalescence, or sedimentation	Body lotions, skin creams, balsams, salves, and gels	Cosmeceutical Vitamin A, D, E, K—Vitalipid	[16,64]
					Moisturizer	Nano Emulsion Multi-PeptideMoisturizer—Hanacure	
10.	Nanocrystal	↑ Drug solubility, particle distribution, adhesiveness, dissolution rate, skin penetration of poorly water-soluble drugs	Possibility of aggregation, not appropriate for aqueous APIs, only stable to a certain extent	100% drug loading ability	Moisturizer	Nano-In Hand and Nail Moisturizing Serum and Foot Moisturizing Serum—Nano-Infinity Nanotech	[21,36,65,66,67]
					Toothpaste	Nano WhiteningToothpaste—Whitewash	

**Table 3:** List of patents related to cosmeceuticals.

S. No.	Patent No.	Country	Title	Application	Proof of Concept
1.	CN100386064C	China	Biological wave nano-bioactive skin protection product	↑ Microcirculation of the skin, hence ↑ metabolism and activation of cells, thereby improving the quality and activity of the skin, wherein vitamin E and ginsenosides can prevent skin aging and nourish the skin with good freckle removal effects. Prevents skin aging, nourishes the skin, and has ultraviolet resistance capacity.	A biological wave nano-bioactive skin protectant product comprising nanoparticles of ZrO_2_ and ZnO, vitamin E, and biological wave functional materials, such as ginsenosides and bioactive materials
2.	KR101224378B1	South Korea	Composite Pigment for cosmetic compositions and manufacturing method and manufacturing apparatus thereof	Complexing nanosized pigment particles (shell particles) to the surface of the extender pigment (core particles) to prevent reaggregation of nanosized pigment particles as shell particles and absorption into the human body	A composite pigment for cosmetics and a method for its manufacturing, wherein the composite pigment for cosmetics is coated with shell particles by physical pressure on the surface of the core particles
3.	CN106660812A	China	Porous silica particles, a method for producing same, and cosmetic compounded with same	Porous silica particles in a cosmetic formulation act as a texture enhancer	This provides porous silica particles with a small specific surface area and a large pore volume, provides a method for producing the particles, and provides a cosmetic in which porous silica particles are present
4.	BR102015012999A2	Brazil	Composition, the process of preparation and use of nanocosmetic based on arnauba wax and quercetin with moisturizing, antioxidant and photoprotective action	A nanoparticle of carnauba wax lipid incorporating quercetin with 3-fold higher effectiveness as moisturizer, photoprotector, and antioxidant	The present invention describes a composition and process for the preparation and use of nanocosmetics consisting of lipid nanoparticles formulated with carnauba wax and quercetin incorporated into cosmetic formulations in gel, cream, lotion, or gel–cream forms
5.	KR101578466B1	South Korea	Porous sphere type zinc oxide powder of nanosize, manufacturing method thereof and color cosmetic composition using the same	Provides a spherical porous zinc oxide powder having a uniform particle size which ↑ the use by ↓ the opacity of the powder	The present invention relates to a spherical porous zinc oxide powder at the nano-scale, a process for its production, and a color cosmetic composition containing the same
6.	KR20120091509A	South Korea	Nano-emulsion containing niacinamide and cosmetic composition comprising the same	A cosmetic composition containing niacinamide-containing nanoemulsions is provided to ↑ the transdermal absorption of niacinamide and to effectively and safely treat dry skin	Associated with niacinamide-containing nanoemulsion and a cosmetic formulation comprising the same
7.	KR101528741B1	South Korea	Silica-containing complex nanoparticles and hydrogel moisturizing patches comprising the same	Silica/zwitterionic polymer complex nanoparticles are able to strongly bind to moisture and accordingly ↓ vaporization speed, thereby having the effects of maintaining moisture and reinforcing the skin barrier	A silica/zwitterionic polymer complex nanoparticle, a manufacturing method thereof, and a hydrogel moisturizing patch
8.	US9700042B2	USA	Nanoformulation of musk-derived bioactive ingredients for nanocosmetic applications	Nanoformulation is applicable to cosmetic and textile manufacturing for providing fragrance and antimicrobial properties in cosmetic and textile products	Nanocarrier composition consists of hyaluronic acid (15–25%) and fatty acids (50–70%) cross-linked with ultra-low-molecular-weight chitosan (15–25%) incorporating isolated compounds from musk and their combinations
9.	CN102274129A	China	Nano-sized core-shell composite material used for cosmetics and preparation method thereof	The composite material has sun-screening and moisturizing functions and dispersibility	The invention comprises a nanosized core–shell composite material composed of titanium oxide and zinc oxide based on the integration of characteristics of the 2 compounds, belonging to the field of skincare cosmetic chemicals
10.	BR102015021346B1	Brazil	Anti-inflammatory, healing and moisturizing tropic cosmeceutical formulation with active ingredients from Atallea Speciosa mart. Ex spreng (Babacu)	Provides anti-inflammatory, healing, and moisturizing activity and may be an alternative and/or therapeutic complement in the treatment of inflammation, tissue healing, and skin hydration processes	Topical anti-inflammatory, healing, and moisturizing cosmetic formulation with active ingredients of *Atallea speciosa* Mart. ex Spreng (babaçu) as plant bioactive compounds containing standardized mesocarp extract and almond oil capable of providing anti-inflammatory, healing, and moisturizing activity
11.	TW201143840A	Taiwan	Compositions and methods for providing ultraviolet radiation protection	Provides excellent UV protection	Sunscreen compositions and related methods that can include a cosmetically acceptable carrier and a multitude of nanoparticles dispersed in the carrier
12.	KR20120058795A	South Korea	Cosmetic composition containing carbon dioxide with nanopore	Provides excellent UV protection	Contains titanium dioxide to ensure high adhesion to the skin and excellent UV protection ability
13.	KR101191268B1	South Korea	Capsule composition contained nano inorganic particles for sunscreen product by hydrogel-forming polymers and manufacturing method thereof	Used to prevent skin penetration of nano-inorganic particles, as deep tissue penetration of nanoparticles results in various types of toxicity	Contains nano-inorganic particles providing UV protection and a method for manufacturing to form a thin hydrogel film
14.	CN108401417A	China	Including improving the cosmetics of nano-particles and preparation method thereof of active principle containing whitening	Excellent nanoparticle for ↑ active principle containing whitening with long-term stability and cutaneous permeation of active principle	A cosmetic incorporating nanoparticles for ↑ active principle with skin-whitening effects; in more detail, the composition functions by ↑ the nanoparticle solubility to hydrophobic whitening active principle via micellization
15.	CN104958189B	China	Light-sensitive color-matching makeup-removal-preventing nanopowder composition and application thereof in cosmetics	The novel skincare product can selectively reflect or scatter external light, only allows skin color light to enter skin, modifies dark skin and uneven surface shadows, and enables the skin to be bright and glossy	A light-sensitive color-matching makeup-removal-preventing nanopowder composition and a preparation method of the composition in cosmetics and application in cosmetics
16.	KR20190085395A	South Korea	Patch composition comprising dog bone gold nano rod, graphene oxide or charcoal	The patch composition can be used as a patch, gel mask, and mask pack with excellent drug delivery into the skin and serves the function of causing an exothermic reaction when activated by LED light source having a wavelength of 700–1200 nm in the near-infrared region	A patch composition containing dog bone gold nanorod or charcoal or graphene oxide having ↑ visible light absorption; it can be used as a mask pack, having the effect of ↑ the drug delivery efficiency into the skin by implementing the target photothermal effect temperature of 41–45 °C by using an exothermic reaction
17.	CN107001774B	China	Positive spherical monodisperse nanoparticle polyester resin water system dispersion and manufacturing method, positive spherical monodisperse nanoparticle polyester resin particle and cosmetics	Provides a kind of cosmetic that has good ductility in which there is good water resistance, softening skin	A novel technical method that is simple and inexpensive, steadily obtains useful positive spherical monodispersed nano-particle polyester resin particles and and water system dispersion
18.	CN102958505B	China	Nanofiber laminate sheet	Appropriately used as a sheet-shaped make-up cosmetic	A nanofiber laminate sheet consisting of a layer of nanofibers composed of a water-insoluble polymeric compound, and a layer of a water-soluble polymeric compound includes a cosmetic component/a medicinal component
19.	KR20130134580A	South Korea	Cosmetic compositions and layer comprising ultra-thin carbon	The thin-layer-laminated structure forms a coating film of the cosmetic composition on the skin, thereby having structural effects of making the active ingredients of the cosmetic composition useful for a long time and exhibiting excellent physical properties through a synergy effect	A cosmetic composition containing an ultra-thin carbon material having a surface diameter of 5–50 μm, which is a plate-type material made from graphite and has 1–10 molecular layers.
20.	KR20140030395A	South Korea	The sunblock through hybrid of nanoparticle of a metal compound, the process for producing thereof, and the cosmetic utilizing thereof	Excellent UV protection	A sunblock agent formed by hybridization of nanoparticles of a metal compound, a process for producing the same, and cosmetic products utilizing the sunblock. More specifically, an organic and inorganic hybrid sunblock causes organic and inorganic hybridization by binding metal oxide nanoparticles of TiO_2_ or ZnO with one or more types of silane
21.	TW200846027A	Taiwan	Nanocomposite pigments in a topical cosmetic application	↑ Aesthetics and skin appearance	Introduction and the preparation of nano-pigments, with their role in ↑ aesthetic properties and skin appearance
22.	US20100003291A1	USA	Nano-particles for cosmetic applications	↑ Characteristics of nanocosmetics and nanocosmeceuticals	Nanocosmetic and nanocosmeceutical preparations and their role in ↑ characteristics by improving the shortcomings of the traditional cosmetic preparation
23.	CN101909580A	China	The Nanoparticulate compositions of enhanced color are provided to cosmetic formulations	↑ Aesthetic properties, specifically color and skin appearance	The pharmaceutical composition comprises 1 or more pigments and the method for ↑ the external appearance of the biological surface by the optical characteristics
24.	TW200533379A	Taiwan	Healthcare and cosmetic compositions containing nanodiamond	↑ Mechanical strength of the cosmetic formulation	Shows ↑ binding capacity with the biological system and thus ↑ its mechanical strength; used in a variety of cosmetic formulations such as shampoo, nail polish, deodorants, eyeliners, etc.
25.	KR20120058795A	South Korea	Cosmetic composition containing carbon dioxide with nanopore	Outstanding UV skin protectant due to good adhesive properties	Formulation containing 0.1–10 wt % TiO_2_ having a size in the range of 200–500 nm, providing protection against UV radiation and making the formulation softer
27.	CA3124455A1	Canada	Microparticles of cellulose nanocrystals with pigment nanoparticles bound thereto and method of production thereof	Preparation of several nanocosmeceuticals and nanocosmetics	Microparticles are formed by clustering nanocrystals and nanoparticle pigments, which are absorbed on the surface of nanocrystals
28.	CN102112100B	China	Preparation of cationic nanoparticles and personal care compositions comprising said nanoparticles	Used as an antimicrobial agent in the cosmetic preparation	Utilization of cationic nanoparticles in the cosmetic formulation and their method of preparation and applications
29.	KR100740275B1	South Korea	Method for preparing zinc oxide powder with nanosize	UV protectant with ↑ transparency and adhesiveness	Method of preparation of nano-range ZnO particles by one-step wet preparation and their application in cosmetics
30.	KR100785484B1	South Korea	Base composition encapsulating high concentration of idebenone with nano sizes, its manufacturing method thereof, and cosmetic compositions containing it	Easily alter the viscosity of the cosmetic and hence ↑ the efficiency of production with ↓ cost	Method of preparation and application of the nanoencapsulation of ↑ concentration of idebenone in a bioactive base for cosmetics production
31.	WO2021144889A1	WIPO (PCT)	Nanobubble-containing cosmetic	Preparation of stable nanobubble solution	Introduction of nanobubble solution in the cosmetic formulation as an active ingredient
32.	KR101436540B1	South Korea	UV protection cosmetic composition comprising titania nanorod	UV protectant and transparency in cosmetics	Synthesis of a cosmetic formulation comprising titania nanorods as a potential sun protectant
33.	CN102397168B	China	Flexible nanoliposomes with charges for cosmetics and preparation method thereof	↑ Stability, permeability, efficiency, retention time, and action of the active ingredients	Introduction to flexible nanoliposomes and their utilization in the cosmetic formulations

**Table 4:** Comparison of regulation of cosmetics/cosmeceuticals in the USA, the European Union, and India [7].

Country	Regulatory Authority	Rules	Approval (Premarket)	Labeling	Labeling Declarations	Language of Label	Expiry Date	Safety	Warning
USA	USFDA	Food, Drug, and Cosmetic Act	No specific requirement	Must conform with the FP&L and FD&C	21 CFR 701 and 740 of USFDA	English	Not required	Manufacturer responsibility	On the primary display panel
EU	EMEA	Council Directive 76/768/EEC	No specific requirement	Based on Council Directive	Cosmetic Directive, Article 6	National or member state	If the stability is <30 months → Date of minimum stability is mentioned; If stability is >30 months → days/months/years after opening is mentioned	Information file of the product is being maintained by the manufacturer	On both outer and inner label
India	CDSCO	Drugs and Cosmetics Act, 1940	Required under the state government licensing	Comply with D&C rules 1945—Part XV	BIS and PCRO	English	It should have “Use before date”	The records of the product’s safety must be maintained by the manufacturer	On inner label

## Data Availability

Not applicable.
